# Blackleg in cattle in Kazakhstan: regional epizootology, seasonal patterns, and molecular identification of the pathogen

**DOI:** 10.3389/fvets.2025.1680881

**Published:** 2025-10-08

**Authors:** Assilbek Mussoyev, Aspen Abutalip, Ainur Nurpeisova, Vladislava Suchshikh, Yerkebulan Makulbekov, Han Sang Yoo, Akmaral Adambayeva, Kanat Kalkabayev, Nurkuisa Rametov, Marhabat Kassenov, Zhandos Abay

**Affiliations:** ^1^Faculty of Veterinary Science, Kazakh National Agrarian Research University, Almaty, Kazakhstan; ^2^Kazakh Scientific Research Veterinary Institute, Almaty, Kazakhstan; ^3^Shakarim University, Semey, Kazakhstan; ^4^College of Veterinary Medicine, Seoul National University, Seoul, Republic of Korea; ^5^National Veterinary Reference Center, Almaty, Kazakhstan; ^6^Department of Geospatial Engineering, Satpaуev Kazakh National Research Technical University, Almaty, Kazakhstan

**Keywords:** blackleg, *Clostridium chauvoei*, cattle, epizootiology, molecular identification, phylogenetic analysis

## Abstract

**Introduction:**

Blackleg (emphysematous carbuncle) is a highly lethal infection of cattle and remains a significant veterinary concern in Kazakhstan. Understanding epidemiological patterns and characterizing the causative agent are crucial for improving surveillance and prevention strategies.

**Methods:**

We analyzed blackleg outbreaks in Kazakhstan from 2015 to 2024 and conducted laboratory investigations on suspected cases in 2023. Isolation and identification of the pathogen were carried out using cultural, morphological, and molecular methods, and phylogenetic analysis was performed based on the 23S rRNA gene fragment.

**Results:**

During 2015–2024, 12–81 outbreaks were reported annually, with an average of 24.6 ± 8.9 outbreaks per year (range 12–81) and 1–3 affected animals per outbreak. A pronounced seasonal peak occurred in November (32.2%). Regional differences were significant: the highest proportion of outbreaks was in West Kazakhstan (55.5%), while low rates were recorded in North Kazakhstan (0.7%) and Akmola (2.1%). Clinical and pathological changes corresponded to classical signs of blackleg. *Clostridium chauvoei* was isolated and identified from two cases in West Kazakhstan and Abai regions. Cultural, morphological, and biological studies confirmed the diagnosis, and phylogenetic analysis verified species identity and provided new genetic data for regional strains.

**Conclusion:**

These findings enhance the understanding of blackleg epizootiology in Kazakhstan and contribute to the establishment of a regional genetic database of *Clostridium chauvoei*, supporting improved surveillance, as well as the development of diagnostic and preventive strategies.

## 1 Introduction

Many species of *Clostridium* cause diseases of public health importance, primarily due to their production of toxins. These protein toxins are responsible for the specific clinical manifestations characteristic of individual clostridial infections. The most well-known examples include *Clostridium difficile* (*C. difficile*) – pseudomembranous colitis, *Clostridium tetani* (*C. tetani*) – spastic paralysis (tetanus), and *Clostridium botulinum* (*C. botulinum*) – flaccid paralysis (botulism). Other members of the genus *Clostridium*, although producing a variety of toxins, may cause diseases with similar clinical presentations. For instance, myonecrosis can be caused by individual species or their combinations, such as *Clostridium septicum* (*C. septicum*), *Clostridium chauvoei* (*C. chauvoei*), *Clostridium novyi* (*C. novyi*) type A, *Clostridium perfringens* (*C. perfringens*) type A, and *Clostridium sordellii* (*C. sordellii*). The morphology of lesions is often similar regardless of the etiological agent (1, 2). *C. septicum* is one of the pathogens involved in the gas gangrene complex and is very similar to *C. chauvoei* (3).

Blackleg (also referred to as emphysematous carbuncle or gas gangrene) is caused by *C. chauvoei*, an anaerobic, motile, Gram-positive, soil-borne bacterium capable of inducing fatal disease in cattle and sheep. The pathogen was named after Auguste Chauveau, a French bacteriologist and veterinarian (4). This acute infectious disease affects the musculature of animals, leading to gangrenous inflammation with gas (emphysema) formation in tissues. It progresses rapidly and often results in the death of the affected animal if urgent treatment is not provided (3, 5). In cattle, characteristic lesions of emphysematous swelling of the musculature often develop without a history of wounds, whereas in sheep the disease is almost always associated with wound infections following shearing cuts, docking, castration, and similar procedures. The disease is highly fatal (2). *C. chauvoei* can also infect buffaloes, goats, wild ruminants such as elk and deer, and is particularly dangerous for unvaccinated livestock, while immunized animals show significantly higher resistance (6).

Transmission occurs mainly via ingestion of spores from contaminated soil or vegetation (5, 6). The vegetative form of the bacterium produces toxins that cause severe clinical signs and ultimately host death (7). Pastures contaminated with spores can remain infectious for years, and cases may recur seasonally. Spore persistence in manure and survival through anaerobic digestion processes in biogas plants further increases the risk of environmental recontamination (8).

Blackleg is reported worldwide (9, 10) and has been known since ancient times among pastoral communities. In Kazakhstan, this disease was historically distinguished from anthrax and named “karasan” (black thigh or buttock) (11). At present, blackleg is among the leading causes of acute infectious outbreaks in livestock in Kazakhstan, second only to rabies. Over the last decade, 12 to 81 outbreaks have been reported annually, with recurrent cases in the same regions, indicating persistent infection foci associated with long-term survival of spores in the environment (12). Even a single occurrence creates chronically unfavorable areas where infections are repeatedly recorded, leading to substantial economic losses for livestock producers (13–15).

Vaccination is the main preventive measure against blackleg, but despite widespread use, scientific data on the efficacy of *C. chauvoei* vaccines in reducing morbidity and mortality remain limited (16). Diagnosis traditionally relies on epizootiological data, clinical signs, and pathological changes, but these criteria are not definitive. Confirmation typically requires bacteriological culture and animal inoculation, which may take 5–10 days (11). Modern approaches such as ELISA, PCR, and fluorescent antibody tests (FAT) offer higher sensitivity and specificity (17–20), though their cost and equipment requirements limit implementation in many veterinary services. Serological methods for detecting specific antibodies are of little diagnostic value due to the acute course of the disease, but can be useful for monitoring vaccine responses (11, 12).

Given the high mortality and economic significance of blackleg, there is a clear need for improved surveillance, molecular characterization of circulating strains, and baseline data to guide prevention strategies. In Kazakhstan, despite frequent outbreaks, few studies have combined large-scale epizootiological analysis with laboratory confirmation and molecular identification of the pathogen.

Therefore, the aim of this study was to investigate the epizootiological situation of blackleg in Kazakhstan during 2015–2024, to describe the clinical and pathological features of cases, and to characterize the biological and molecular properties of the isolated pathogen. The study also seeks to provide baseline genetic data to support improved surveillance and future development of diagnostic and preventive strategies.

## 2 Methods

### 2.1 Epizootiological analysis

The epizootiological situation of blackleg in cattle was analyzed using official veterinary reports of the Republic of Kazakhstan for the period 2015–2024. The analysis included the number of registered outbreaks, their distribution by regions and years, and the average outbreak index (the mean number of affected animals per outbreak). The seasonality of the epizootic process was assessed based on the monthly distribution of cases for the period 2020–2024.

**Figure 1:**
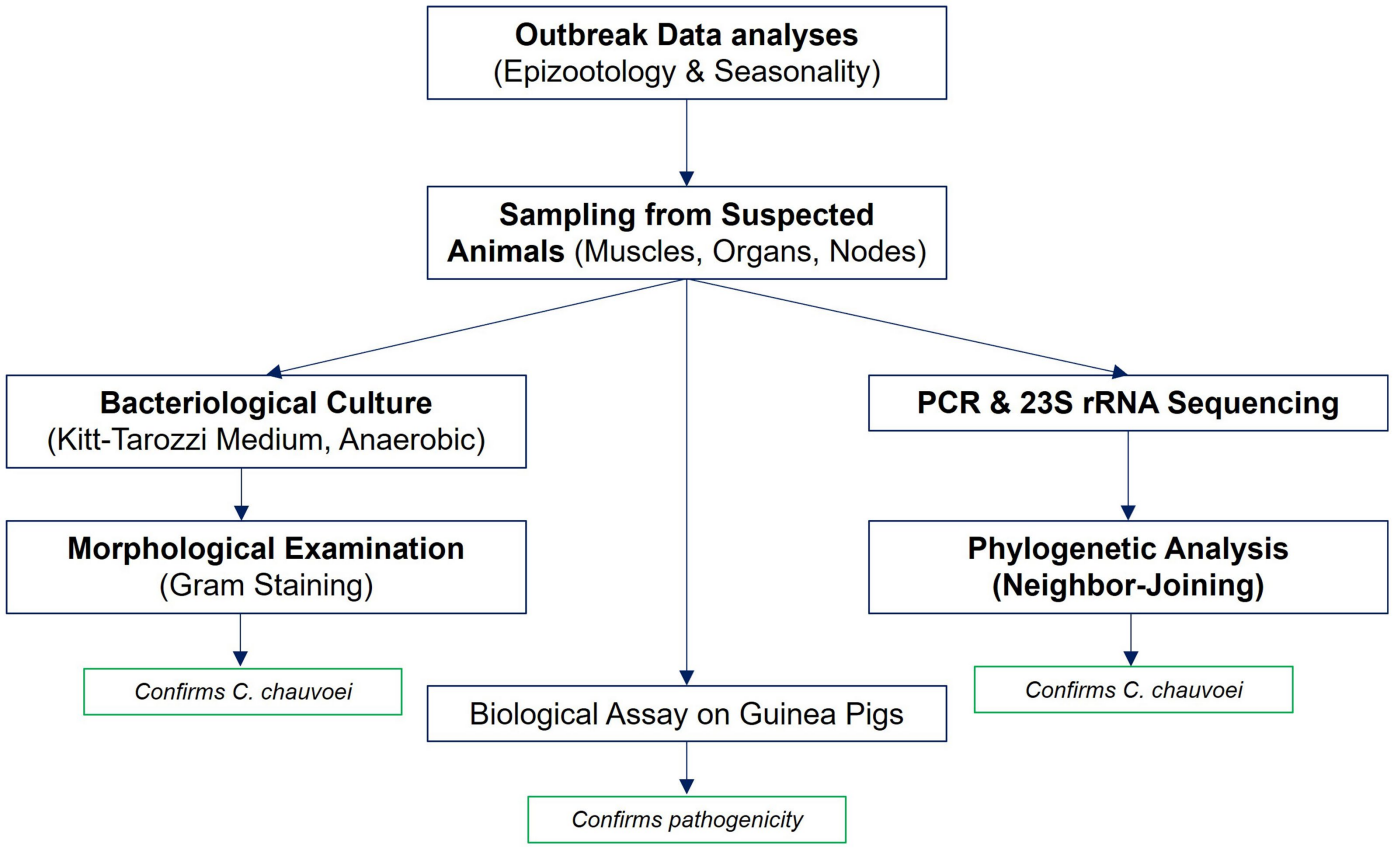
Workflow of the study. The workflow illustrates the main stages of the study: analysis of outbreak data (epizootological and seasonal assessment), sampling of pathological material from suspected cattle, bacteriological culture on Kitt-Tarozzi medium with Gram staining, molecular identification by PCR and 23S rRNA sequencing with subsequent phylogenetic analysis and biological assay on guinea pigs.

The sequence of study stages, from outbreak data analysis to molecular identification and biological assay, is presented in Figure 1.

### 2.2 Sampling of pathological material and bacteriological studies

From 33 registered outbreaks of blackleg in cattle in 2023, pathological material was collected from nine animals suspected or confirmed to have blackleg in West Kazakhstan, Abai, Pavlodar and Karaganda regions. Selection criteria included sudden death, subcutaneous emphysema, and dark-red swollen musculature observed during postmortem examination and verified by veterinary reports from the respective outbreak sites. Samples included affected muscles, lymph nodes, and parenchymal organs.

Sampling and transportation were performed in accordance with biosafety protocols. Pathological material was packaged in triple-containment systems, transported under BSL-2 conditions, and processed under BSL-3 containment in compliance with OIE biosafety guidelines. For pathogen isolation, samples were cultured under strict anaerobic conditions on Kitt-Tarozzi medium and other nutrient media recommended for *Clostridium* spp. Gram staining was performed to assess morphology, confirming Gram-positive, spore-forming rods characteristic of *C. chauvoei*.

### 2.3 Field data collection and mapping

Field data were collected using Survey123 for ArcGIS, and spatial analysis with cartographic visualization was performed in ArcGIS Pro 3.4 (Esri Inc., Redlands, CA, USA). Final maps were prepared using standardized layouts and exported at high resolution in the WGS 1984 UTM coordinate system.

### 2.4 Biological assay

To confirm the pathogenicity of the isolated *C. chauvoei* cultures, a biological assay was performed in guinea pigs. Two groups of animals (*n* = 3 per group) were inoculated intramuscularly with 0.1 mL of a pre-titrated suspension of each isolate at a dose of 20 DLM (LD_50_). Group 1 received the Abai isolate (#1), and Group 2 received the West Kazakhstan isolate (#2). Animals were monitored for 5 days for clinical signs and pathological changes.

The use of six guinea pigs in total (*n* = 3 per isolate) was based on the ethical principles of the 3Rs (replacement, reduction, refinement) and OIE recommendations for laboratory confirmation, where the minimal number of animals is justified by diagnostic rather than experimental objectives as described in WOAH Terrestrial Manual 2021, The Merck Veterinary Manual 2023 and OIE 3Rs 2019.

### 2.5 Molecular identification

Total DNA was extracted using the PureLink Genomic DNA Mini Kit (Invitrogen, USA) according to the manufacturer’s instructions. DNA quality and quantity were assessed by agarose gel electrophoresis, spectrophotometry (NanoQuant, Tecan), and fluorometry (Qubit 3.0, Invitrogen). Sequencing libraries were prepared with the Nextera XT DNA Sample Preparation Kit (Illumina, USA) and sequenced on an Illumina MiSeq platform (2 × 300 bp). Raw reads were quality-checked (FastQC), trimmed (Trimmomatic v0.36), and assembled in Geneious. The 23S rRNA gene was identified using BLASTn and aligned with reference sequences (NCBI GenBank) using MAFFT. A phylogenetic tree was constructed in Geneious using the neighbor-joining method with 1,000 bootstrap replicates.

### 2.6 Statistical analysis

Descriptive statistics were used to summarize outbreak data. Regional and temporal outbreak frequencies were compared using Pearson’s chi-square test (χ^2^). Linear regression was applied to assess temporal trends in the number of outbreaks during 2015–2024. Seasonal variation was analyzed using chi-square tests with Bonferroni correction for multiple comparisons. Ninety-five percent confidence intervals (95% CIs) for proportions were calculated using Wilson’s method. Seasonality was evaluated based on the monthly distribution of outbreaks for the period 2020–2024. All statistical analyses were performed in Microsoft Excel 2016 (Microsoft Corp., Redmond, WA, USA).

### 2.7 Ethical considerations

All procedures involving laboratory animals were conducted in accordance with the OIE principles of humane treatment and were approved by the Local Ethics Committee of the Kazakh Scientific Research Veterinary Institute (Protocol No. 11, 10 December 2023).

## 3 Results

### 3.1 General epizootiological characteristics of blackleg in the Republic of Kazakhstan

To assess the epizootiological situation of blackleg in cattle in the Republic of Kazakhstan, a comprehensive analysis of data from 2015 to 2024 was conducted. The study included information from statistical reviews, official veterinary reports, and materials from clinical and epizootiological surveys. Particular attention was given to the quantitative characteristics of epizootic outbreaks, the regional dynamics of morbidity, and the identification of factors potentially influencing the spread of the infection.

During the analyzed period (2015–2024), a total of 1,864 outbreaks of acute infectious animal diseases were registered across the country, of which 421 were attributed to blackleg, representing 22.6% (Table 1).

**Table 1:** Proportion of blackleg outbreaks among all acute infectious animal diseases in Kazakhstan by region for 2015–2024 with 95% confidence intervals (CIs).

Region	Total outbreaks (*N*)	Blackleg outbreaks (*n*)	Proportion, % (95% CI)
West Kazakhstan	254	141	55.5 (49.4–61.5)
East Kazakhstan	249	76	30.5 (25.1–36.5)
Zhambyl	206	42	20.4 (15.5–26.4)
Almaty	134	43	32.1 (24.8–40.4)
Aktobe	116	41	35.3 (27.2–44.4)
Pavlodar	104	28	26.9 (19.3–36.0)
Kostanay	130	6	4.6 (2.0–9.8)
Karaganda	123	13	10.6 (6.2–17.3)
Atyrau	79	6	7.6 (3.5–15.6)
Akmola	97	2	2.1 (0.6–7.3)
North Kazakhstan	143	1	0.7 (0.1–3.8)
Kyzylorda	20	5	25.0 (11.2–46.9)
Mangystau	26	0	0.0 (−)
Turkestan	129	0	0.0 (−)
Zhetysu	12	1	8.3 (1.5–35.4)
Abai	30	7	23.3 (11.9–40.3)
Ulytau	12	0	0.0 (−)

The regional distribution of outbreaks was highly uneven. The highest proportion was observed in the West Kazakhstan region (55.5, 95% CI, 49.4–61.5), whereas the lowest rates were in North Kazakhstan (0.7%) and Akmola (2.1%). Several regions (Turkestan, Mangystau and Ulytau) reported no outbreaks during the analyzed period. Statistical comparison confirmed that differences between regions were highly significant (χ^2^ = 348.0, *p* < 0.0001), highlighting pronounced regional heterogeneity of the epizootiological situation (Table 2).

**Table 2:** Dynamics of blackleg outbreaks among all acute infectious animal diseases in Kazakhstan for 2015–2024 with 95% confidence intervals (CIs).

Year	Total outbreaks (*N*)	Blackleg outbreaks (*n*)	Proportion, % (95% CI)
2015	277	45	16.3 (12.4–21.1)
2016	149	21	14.1 (9.4–20.6)
2017	142	47	33.1 (25.9–41.2)
2018	163	40	24.5 (18.6–31.7)
2019	183	63	34.4 (27.9–41.6)
2020	326	81	24.9 (20.5–29.8)
2021	204	44	21.6 (16.5–27.7)
2022	195	26	13.3 (9.3–18.8)
2023	125	33	26.4 (19.5–34.8)
2024	100	12	12.0 (7.0–19.8)

**Figure 2:**
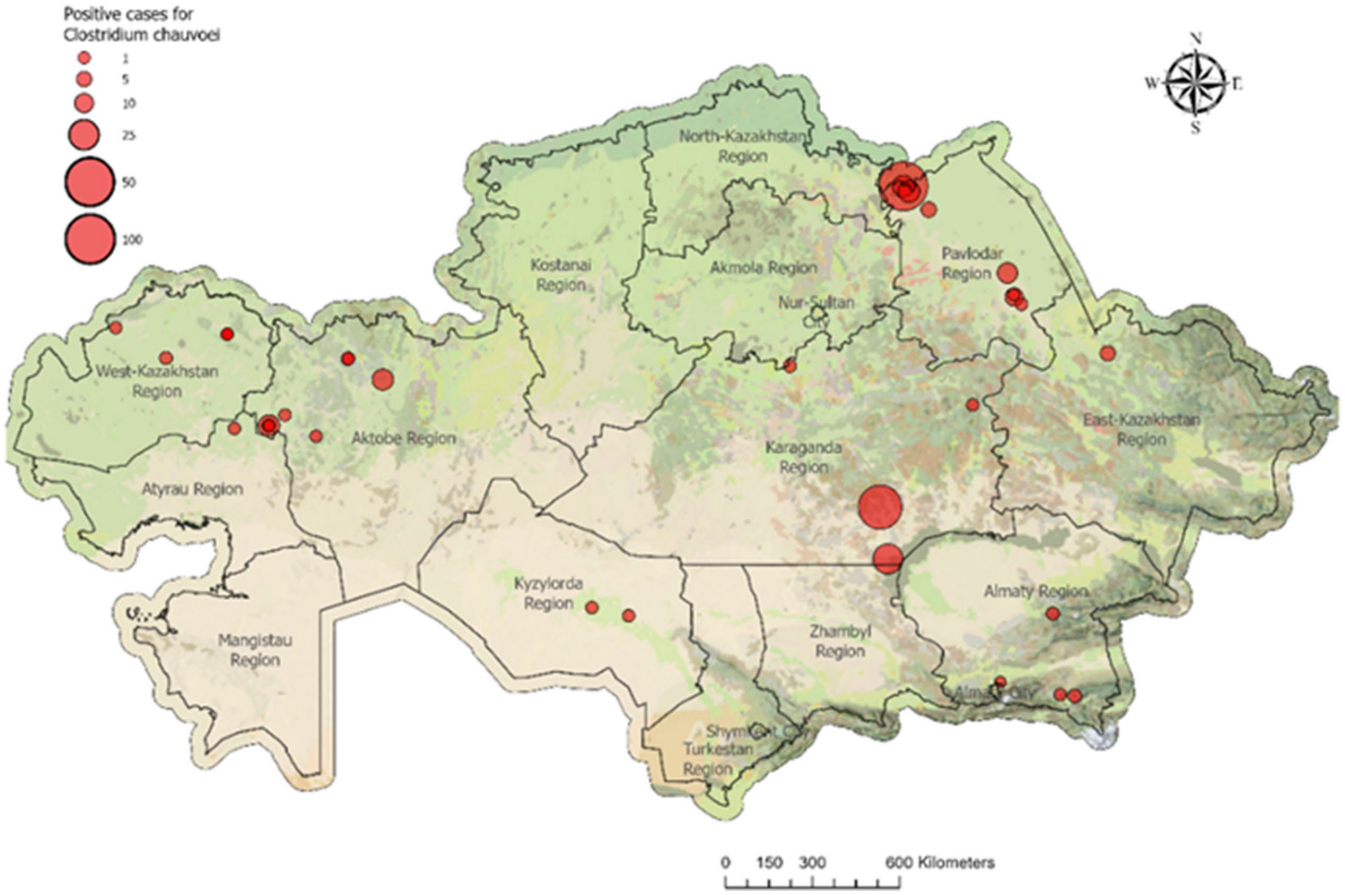
Registered 33 blackleg outbreaks in cattle in 2023. In 2023, 33 blackleg outbreaks were reported in Kazakhstan. Most outbreaks occurred in Pavlodar and Aktobe regions (8 each) and West Kazakhstan region (5). In total, 234 cattle were infected and culled nationwide, with the highest numbers reported in Pavlodar (107), Karaganda (72), and Aktobe (35) regions.

Temporal dynamics of outbreaks in 2015–2024 were variable. The lowest proportion of outbreaks was recorded in 2024 (12.0, 95% CI, 7.0–19.8), while peaks were observed in 2017 (33.1%) and 2019 (34.4%). Linear regression analysis did not reveal a significant long-term trend in outbreak frequency (R^2^ = 0.07, *p* = 0.47), indicating that interannual fluctuations were largely influenced by local epizootiological factors rather than a systematic increase or decrease over time. The outbreak index for blackleg ranged between 1 and 3 animals per outbreak, supporting the non-contagious nature of the disease (Figure 2).

Seasonality analysis revealed a clear autumn–winter predominance. The highest number of outbreaks occurred in November (32.2% of the total), with moderate increases in October and December (7.8% each). The lowest activity was recorded in summer months, particularly in August (3.6%). Chi-square testing demonstrated that the monthly distribution of outbreaks deviated significantly from uniformity (*χ*^2^ = 150.4, *p* < 0.0001), confirming a statistically significant seasonal component in the epizootic process (Table 3).

**Table 3:** Seasonal distribution of blackleg outbreaks in Kazakhstan for 2020–2024 (in absolute numbers and as a percentage of the total).

Month	Jan	Feb	Mar	Apr	May	Jun	Jul	Aug	Sep	Oct	Nov	Dec	Total
2020	4	3	4	3	2	3	2	1	4	5	39	10	81
2021	3	6	4	3	5	5	1	4	4	3	5	1	44
2022	3	1	0	4	2	2	2	2	1	2	2	1	22
2023	1	0	1	2	0	2	1	1	5	3	14	3	33
2024	1	1	0	1	0	1	0	1	1	4	2	0	12
Total	12	11	9	13	9	13	7	9	15	17	62	15	192
%	6.25	5.7	4.6	6.7	4.6	6.7	3.6	4.6	7.8	8.8	32.2	7.8	–

These results underscore the role of climatic and management factors in pathogen activation and highlight the importance of strengthening preventive measures prior to the autumn peak.

### 3.2 Own investigations of the epizootic process

**Figure 3:**
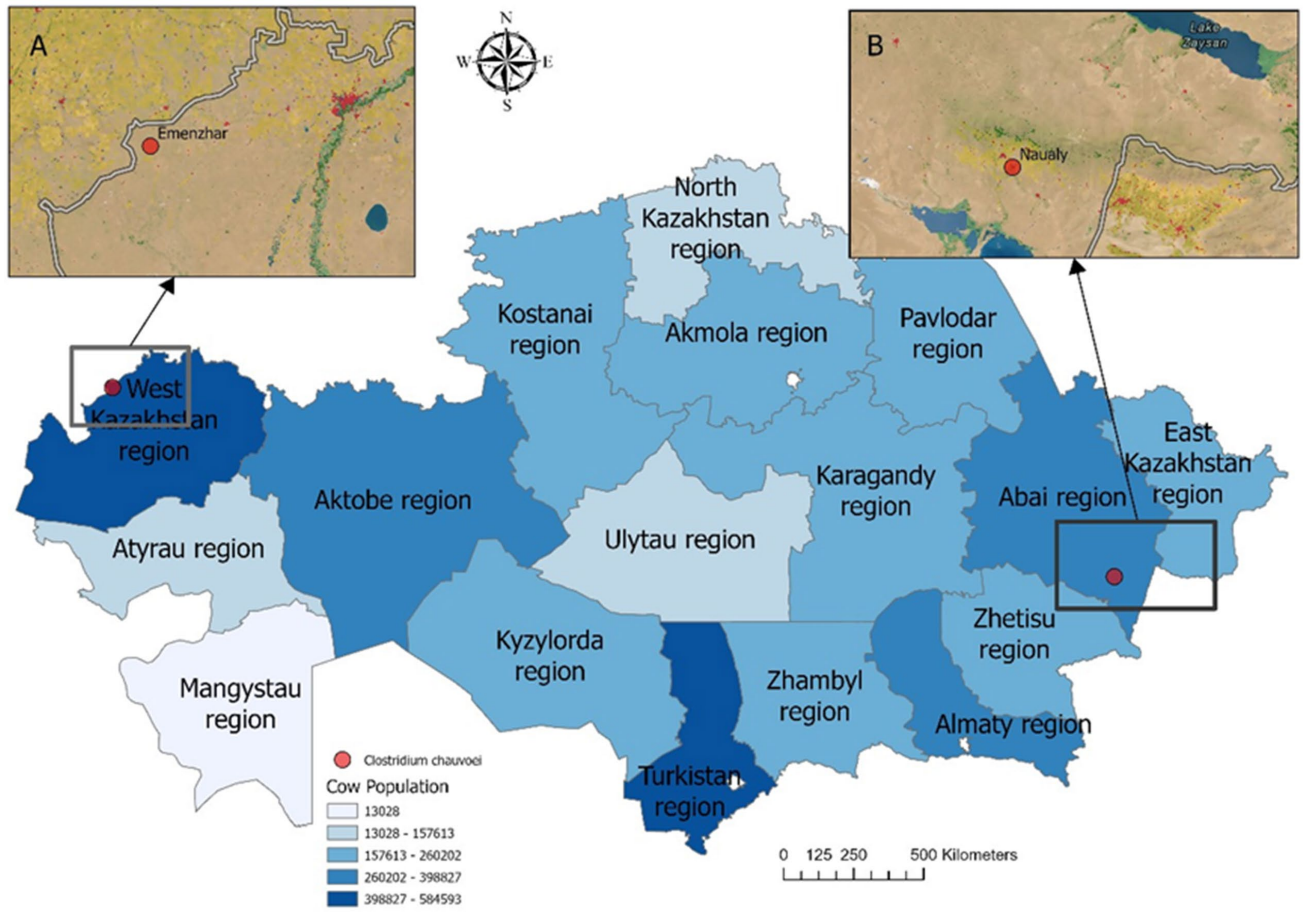
Map of cattle density by localities showing the sampling sites in Kazakhstan. Insets **(A)** and **(B)** highlight specific locations: **(A)** Emenzhar in West Kazakhstan region, **(B)** Naualy in Abai region. Pathogen isolation and laboratory confirmation were achieved in only two of nine sampled animals (red dots: Emenzhar and Naualy). Statistical data for the cattle population in 2025 were obtained from the Bureau of National Statistics of the Republic of Kazakhstan (https://stat.gov.kz). Darker shades correspond to higher cattle density (census data, 2025).

From the 33 registered outbreaks of blackleg in cattle in 2023, pathological material was collected from nine animals showing typical clinical and postmortem signs of the disease in West Kazakhstan, Abai, Pavlodar, and Karaganda regions. In two cases (West Kazakhstan and Abai), isolates with morphological and cultural characteristics consistent with *C. chauvoei* were obtained and subsequently subjected to phenotypic and molecular characterization (Figure 3).

Spatial analysis (Figure 3) confirmed that the isolates were obtained from regions with high cattle density, consistent with the epidemiological distribution of outbreaks.

**Figure 4:**
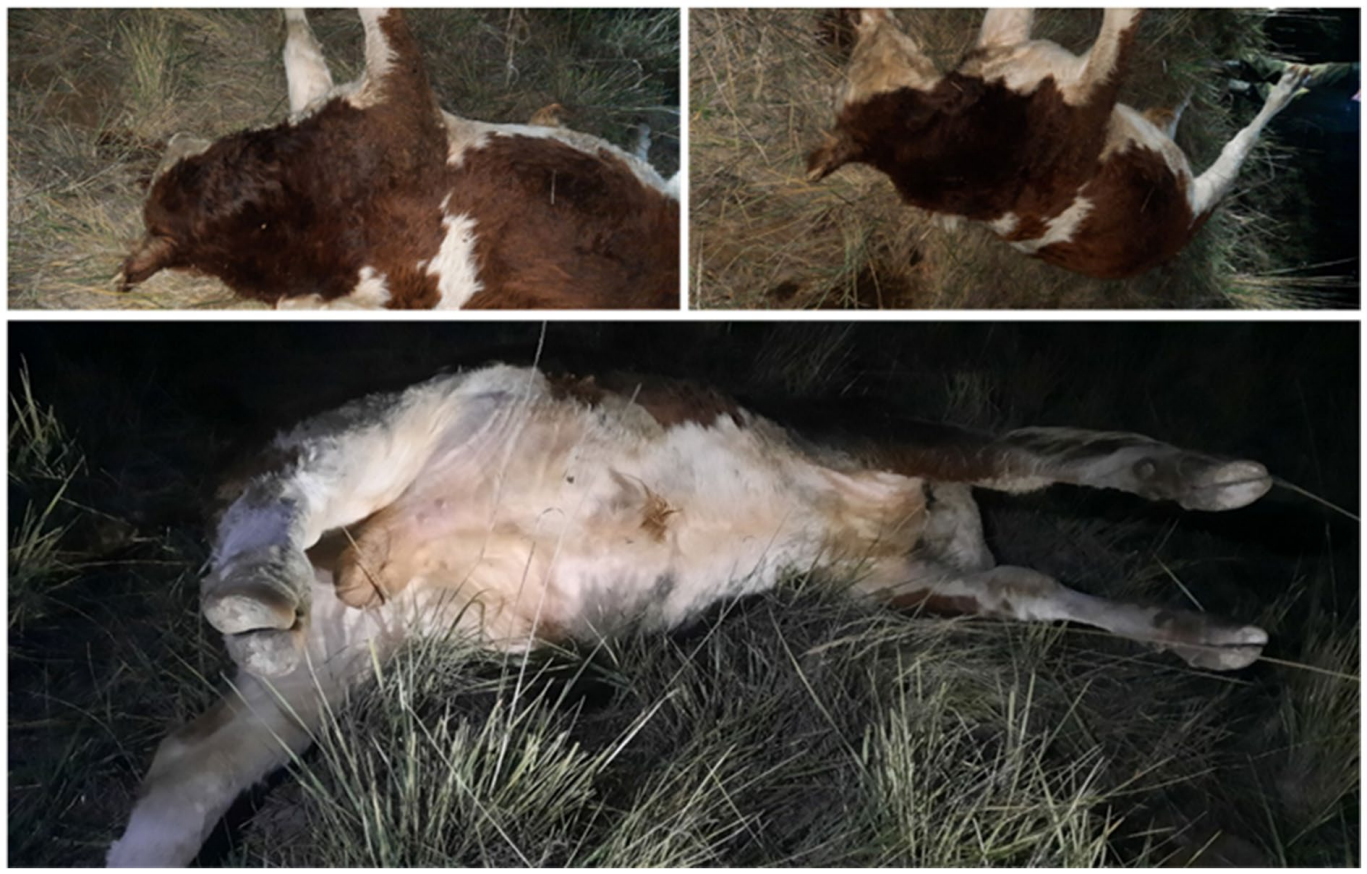
Gross view of cattle carcasses with blackleg showing pronounced subcutaneous edema of the thoracic and abdominal regions, distension of the body and enlargement of the hind limbs.

Clinical observations in affected cattle revealed marked swelling and infiltration of soft tissues in the thoracic and abdominal regions and pelvic limbs, accompanied by generalized carcass bloating (Figure 4).

**Figure 5:**
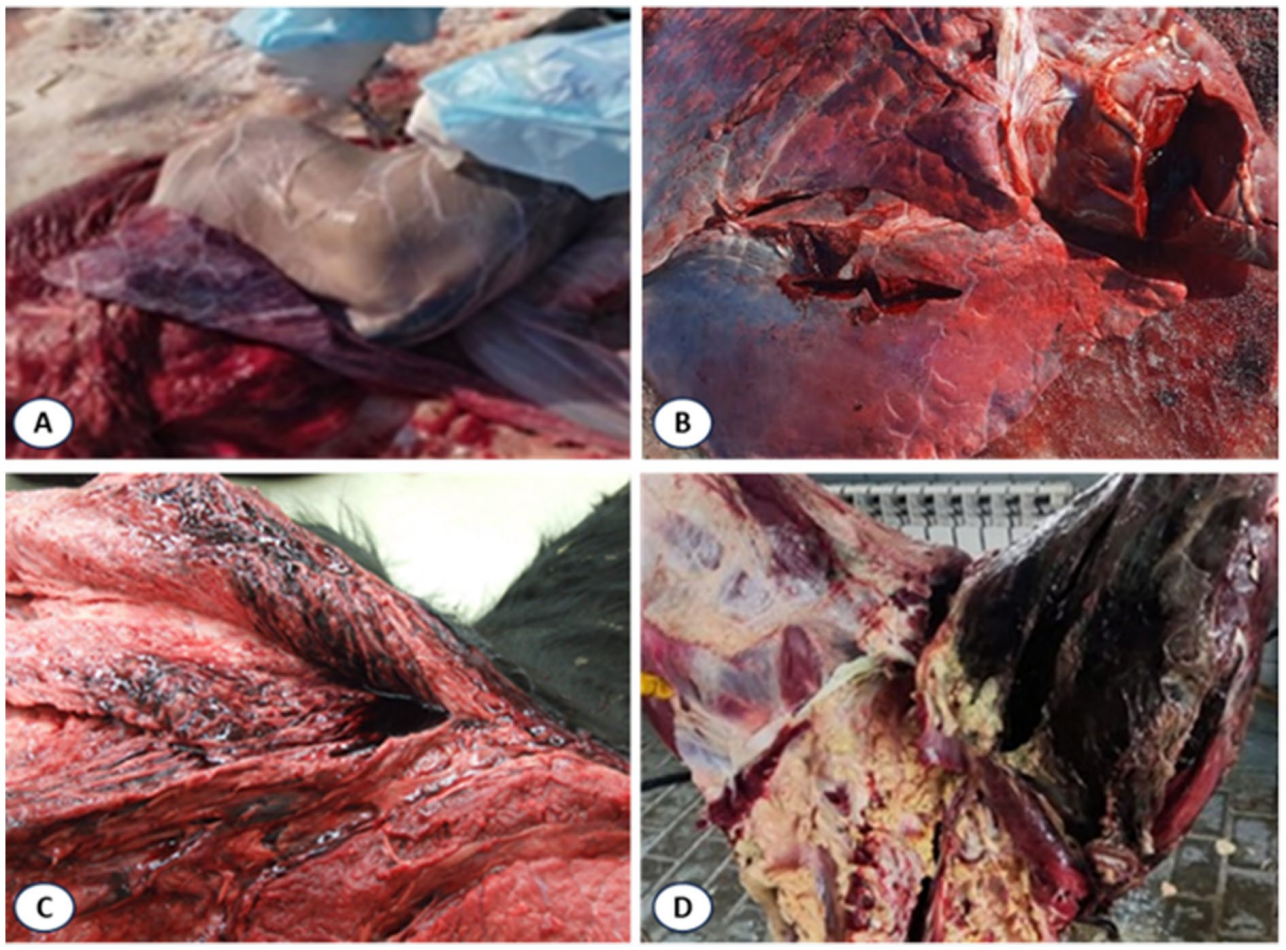
Lesions in muscles of cattle that died of blackleg. **(A)** Swollen spleen; **(B)** Necrotic liver with gas bubbles; **(C)** Dark red to black discolored muscles with gas accumulation; **(D)** Gross lesions in affected tissues.

Necropsy findings included multiple hemorrhages on serosal and mucous membranes, coagulated dark-red blood, and characteristic muscular changes. Expanding carbuncles were present in large muscle groups (rump, loin, thigh, shoulder, neck), showing crepitus on palpation due to gas accumulation. Affected muscles were dark red to nearly black, porous, and dry in consistency, with gas bubbles evident. Splenomegaly with softening and hepatomegaly with necrotic foci and gas inclusions were also noted (Figure 5).

**Figure 6:**
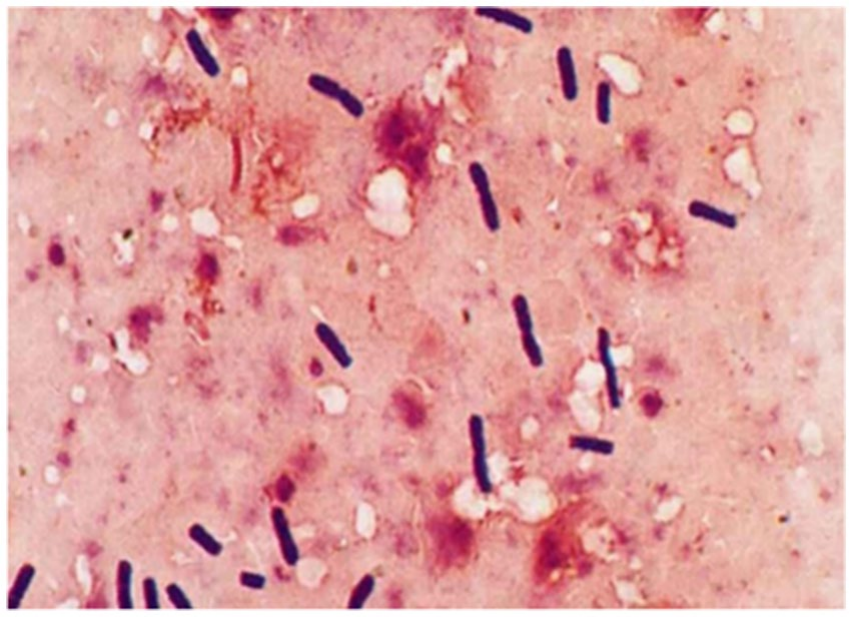
Morphology of the *C. chauvoei* isolate based on Gram staining.

Microscopic examination of the isolated cultures revealed Gram-positive, spore-forming, rod-shaped anaerobes (*C. chauvoei*), measuring 0.5–0.7 × 2–8 μm, motile, non-capsulated, with rounded or slightly curved ends (Figure 6).

**Figure 7:**
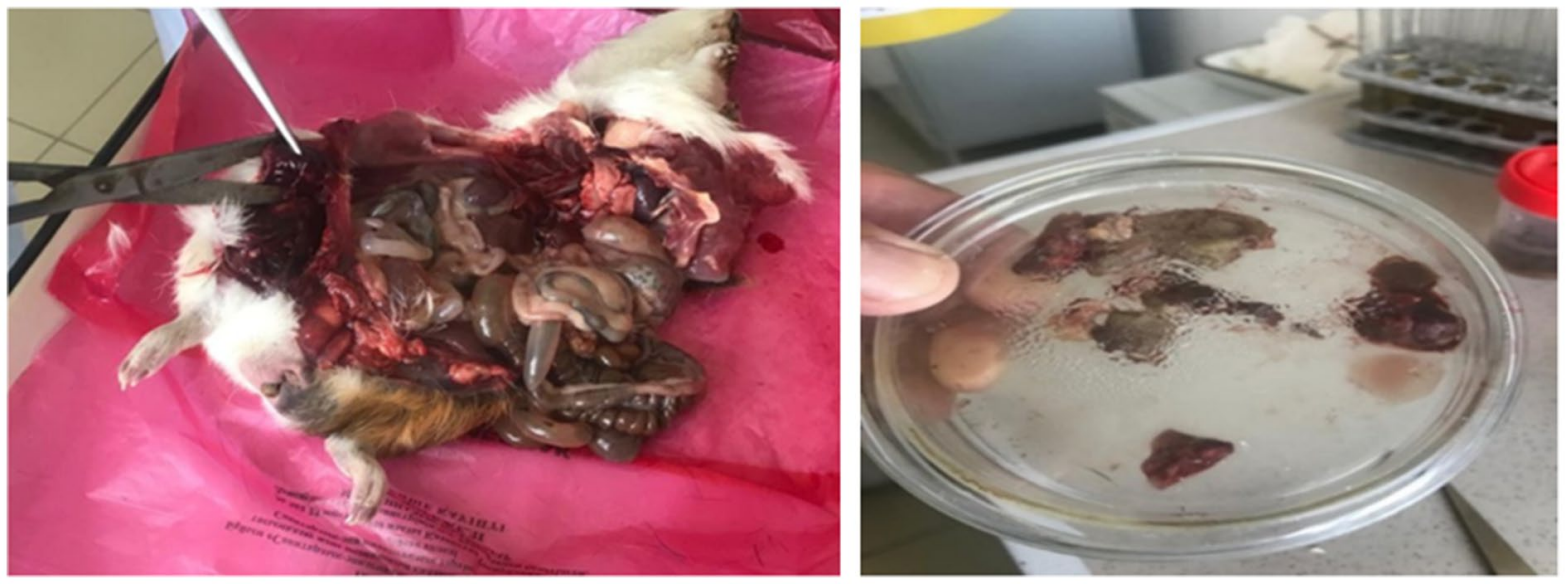
Pathological changes in guinea pigs infected with *C. chauvoei* and tissue sampling for bacteriological analysis.

The guinea pig bioassay revealed marked differences between isolates. In Group 1, all animals succumbed within 24–72 h, showing dullness, depression, lameness, and fever (40.0–41.1°С). Necropsy revealed congestion of the liver and spleen, gas distension of the gastrointestinal tract, blackened muscles surrounded by yellow exudate with gas bubbles, and hemorrhages in enlarged lymph nodes (Figure 7). Bacteriological re-isolation confirmed *C. chauvoei*.

In Group 2, one guinea pig died within 24 h, whereas the remaining two animals remained clinically healthy during the five-day observation period. The isolate from Group 1, which consistently reproduced the disease, was designated *C. chauvoei* strain AsMus-2025 and was used for subsequent molecular analysis.

### 3.3 Sequencing data and genome assembly

The pathogenic isolate from Group 1 was designated *C. chauvoei* strain *AsMus-2025* and subjected to sequencing. Illumina sequencing of the clinical sample yielded 926,152 reads with an average GC content of 57.3%. After quality control, adapter trimming, and filtering using FastQC and Trimmomatic, the reads were assembled into contigs in Geneious.

Due to technical limitations and the small amount of DNA obtained, a complete genome assembly was not achieved. However, one large contig of 200,754 bp was recovered, which included the 23S rRNA gene and enabled species-level analysis and phylogenetic identification. Comparison with reference genomes in GenBank showed 99.98% identity with several *C. chauvoei* strains, including SBP 07/09 (CP027286.1), JF4335 (LT799839.1), 1,250,467 (CP018630.1), and DSM 7528 (CP034512.1). Matches exhibited 100% coverage with E-values near zero, confirming a high degree of homology and reliable species identification.

The obtained nucleotide sequence was deposited in GenBank under accession number PV828315.1 as *C. chauvoei* strain *AsMus-2025.*

### 3.4 Identification of the 23S rRNA gene and phylogenetic analysis

The 23S rRNA gene was identified within the assembled contig using BLAST searches against the NCBI GenBank database. The sequence showed 99.98% identity with *C. chauvoei* reference sequences, confirming the taxonomic identity of the isolate.

**Figure 8:**
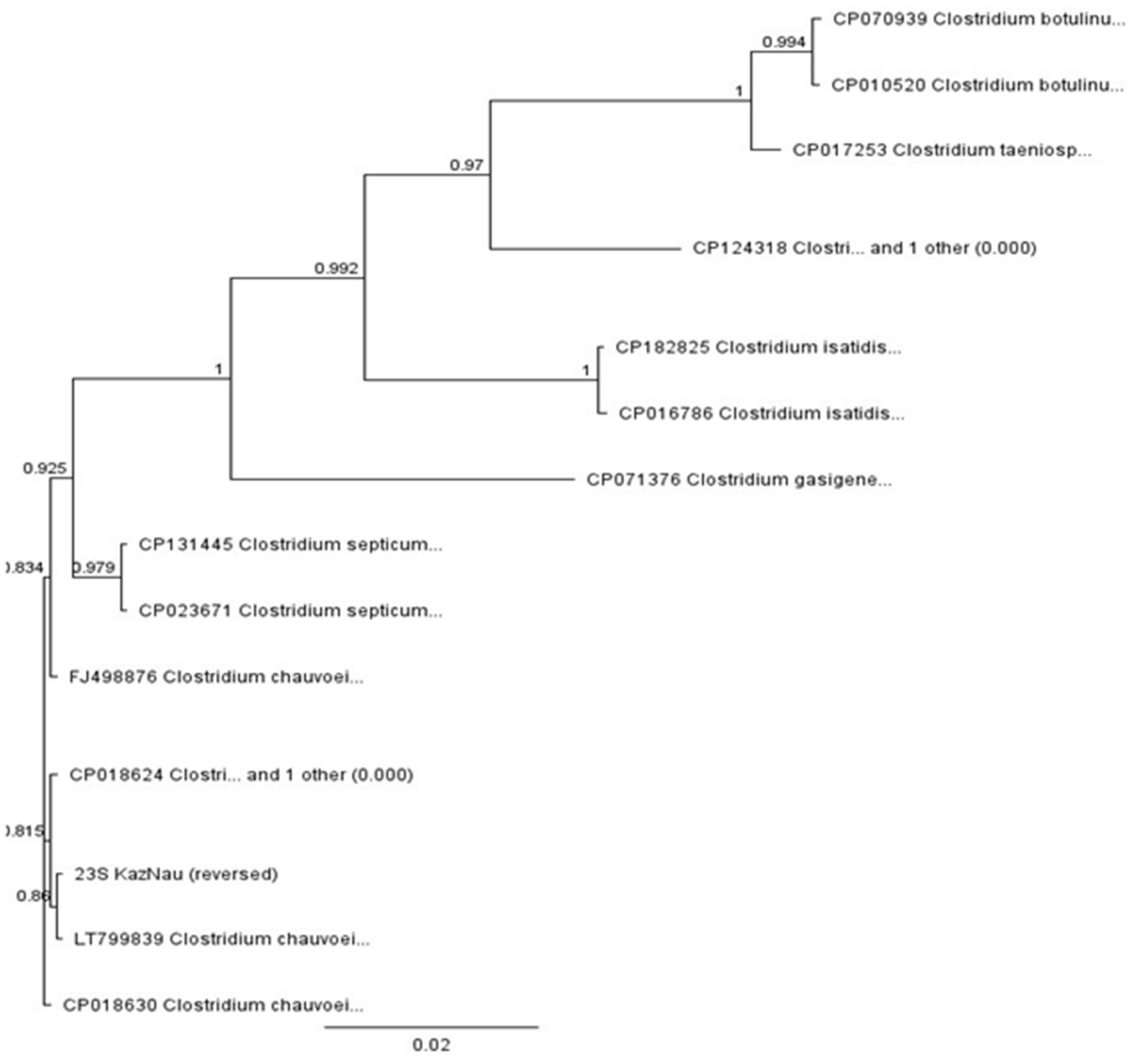
Phylogenetic tree of the genus *Clostridium* based on the 23S rRNA gene fragment.

The gene fragment was aligned with homologous sequences of related *Clostridium* species using MAFFT, and a phylogenetic tree was constructed in Geneious with the neighbor-joining method and 1,000 bootstrap replicates (Figure 8).

The phylogenetic tree (Figure 8) clearly clustered the studied sequence within the *C. chauvoei* clade, together with reference strains LT799839, CP018630, and FJ498876, with strong bootstrap support (>0.98). The isolate was distinctly separated from closely related species such as *C. septicum*, *C. isatidis*, and *C. botulinum*, demonstrating the suitability of the 23S rRNA fragment for interspecies differentiation within the genus.

These results confirm that the sequenced sample represents a circulating *C. chauvoei* strain in Kazakhstan. At the same time, the reliance on a single gene fragment limits conclusions regarding intra-species diversity. Nevertheless, the generated sequence contributes to the establishment of a preliminary local database of *C. chauvoei* genetic variants, providing baseline information for future studies on outbreak monitoring, whole-genome comparisons, and the development of diagnostic assays.

## 4 Discussion

Blackleg remains one of the most dangerous infections affecting cattle and other domestic and wild animals (15, 21, 22). The disease is characterized by severe muscle necrosis with gas formation (crepitant necrosis) caused by active replication of anaerobic bacteria, predominantly *C. chauvoei*. It progresses acutely, with mortality rates ranging from 87 to 100%, and typically results in the rapid death of affected animals (23). Cases of *C. chauvoei* infection have been reported in Iran, Austria, Algeria, Pakistan, Ethiopia, Taiwan, Brazil, Zambia, southeastern Sweden, and Belarus (23, 24).

In Kazakhstan, blackleg is widespread and requires targeted preventive measures, including vaccination, sanitary monitoring, and pasture management in priority regions (25). Our findings confirmed strong regional heterogeneity: the highest burden was observed in West Kazakhstan, East Kazakhstan, Zhambyl, Almaty, and Aktobe regions, while no outbreaks were reported in Mangystau, Turkestan, and Ulytau. Differences across regions were highly significant (χ^2^ = 348.0, *p* < 0.0001). This pattern indicates that ecological conditions, cattle density, and pasture use strongly influence the persistence of infection foci.

Temporal dynamics revealed marked annual fluctuations, with outbreak peaks in 2017 and 2019 and lower numbers in 2022–2024. However, regression analysis did not identify a consistent long-term trend (R^2^ = 0.07, *p* = 0.47), suggesting that interannual variation reflects local epizootiological and management factors rather than a systematic increase or decline.

Seasonality was a prominent feature of the epizootic process. Outbreaks peaked in November (32.2%), with smaller increases in October and December, while the lowest activity occurred in August (3.6%). Statistical analysis confirmed significant deviation from uniformity (*χ*^2^ = 150.4, *p* < 0.0001). These results are consistent with the observations of E. Doğan, Ç. Çolak, and De Jesus Sousa et al. (2, 26, 27), who reported autumn–winter increases linked to grazing practices and climatic triggers. This finding has direct practical relevance: vaccination campaigns should be intensified before the autumn–winter period, and preventive measures should be reinforced in high-risk regions.

The clinical and pathological findings in affected cattle (edema, crepitating carbuncles, bloating of carcasses, muscle necrosis, and foamy exudates) were consistent with descriptions by E. Doğan, Ç. Çolak, and De Jesus Sousa et al. (2, 26, 27), confirming reproducibility across regions. As in previous studies (7, 13), the most severe lesions were in large muscle groups (thigh, rump, loin, shoulder), supporting the role of traumatized tissues as a predisposing factor for disease activation.

Pathogen isolation and laboratory confirmation were achieved in only two of nine sampled animals (West Kazakhstan and Abai). One isolate, designated *C. chauvoei* strain AsMus-2025, was pathogenic in guinea pigs and subjected to sequencing. Molecular studies confirmed its consistency with international reference strains (19, 28). Phylogenetic analysis of the 23S rRNA gene confirmed species identity and grouped the isolate within the *C. chauvoei* clade, consistent with findings of R. C. Guiassa (29), Nicholson et al. (30), and Ziеch et al. (31). While these results provide valuable baseline data, broader comparative genomic analyses are needed to explore intra-species variability and virulence determinants.

Modern diagnostic methods such as PCR, ELISA, and FAT are highly sensitive and specific but remain limited in Kazakhstan due to cost and infrastructure. Our data on molecular identification provide a genetic baseline that may support the future development of rapid and affordable diagnostic solutions. Preliminary work on antigen preparation and immunoassays is ongoing but beyond the scope of this study.

### 4.1 Limitations and future directions

This study was based on officially reported outbreaks, which may underestimate true incidence. Statistical analyses were limited to chi-square and linear regression, without more advanced approaches such as logistic regression. Only nine animals were sampled, with two yielding isolates and one subjected to sequencing, restricting molecular conclusions. The analysis relied on a single genetic marker (23S rRNA), which is suitable for species identification but not for strain-level diversity. Finally, biological assays were performed on small groups of guinea pigs, justified by OIE guidelines and ethical considerations, but still limiting the robustness of pathogenicity assessment (32–34). Another practical limitation is the insufficient production capacity of local immunobiological manufacturers (e.g., SPE Antigen LLP, LLP Biotron), which constrains vaccine availability for the growing cattle population in Kazakhstan. This emphasizes the importance of integrating epidemiological evidence with national vaccine production strategies.

Expanded sampling and whole-genome sequencing are needed to characterize regional strain diversity and virulence determinants. Integration of molecular data with epizootiological mapping could guide the development of region-specific vaccines and more affordable rapid diagnostic kits. Strengthening long-term monitoring and evaluating environmental risk factors will further improve surveillance and outbreak prevention in Kazakhstan.

## 5 Conclusion

This study provides the first comprehensive characterization of blackleg in Kazakhstan over a 10-year period (2015–2024), revealing marked regional heterogeneity and a pronounced seasonal peak in November. The highest incidence was observed in West Kazakhstan, East Kazakhstan, Zhambyl, Almaty and Aktobe regions, whereas several regions reported no cases. Two *C. chauvoei* isolates were recovered from cattle, and one strain (*AsMus-2025*) underwent phenotypic and molecular identification, including 23S rRNA-based phylogenetic analysis, contributing baseline data to a regional genetic database.

These findings have direct practical relevance for veterinary authorities and livestock producers. Vaccination campaigns should be intensified prior to the autumn-winter peak, while continuous monitoring of pastures and chronically affected areas remains essential. The molecular results, though limited in scope, provide a foundation for future genomic studies and may support the development of region-adapted vaccines and more affordable rapid diagnostic tools.

Overall, this work establishes a baseline for epizootiological surveillance of blackleg in Kazakhstan and underlines the importance of integrating epidemiological, molecular, and preventive approaches to reduce the burden of this lethal disease.

## Data Availability

The datasets presented in this study can be found in online repositories. The names of the repository/repositories and accession number can be found at: https://www.ncbi.nlm.nih.gov/genbank/, PV828315.1.
